# Some Suggestions on Life Insurance

**Published:** 1894-05

**Authors:** James L. Hopkins

**Affiliations:** St. Louis Bar.


### SOME SUGGESTIONS ON LIFE INSURANCE.

By JAMES L. HOPKINS,

Of the St. Louis Bar.

Every professional man has given more or less attention to the subject of life insurance. A great part of the incomes of the life companies is derived from them. The writer has been frequently called on for advice regarding insurance, and as many of the inquiries have come from the physicians among his acquaintances, it has occurred to him that a few thoughts on the subject may be acceptable to the clientèle of the *Medical Mirror.*

The first question is, Who needs insurance? The answer is, You do. It matters not that you have no one dependent on you for support, and that you have ample funds to meet all your debts as you go along. The day when the assured had to "die to beat the game" is past. Every company of any standing issues policies with an investment feature, and the returns offered for the use of your money are as good as any ordinary business investment. The man who is without insurance on his life is as foolish as the merchant who fails to insure his stock in trade from loss by fire. This is particularly true of the professional man, whose brains are his sole merchandise. The amount he carries should be regulated solely by his means and ability to pay the premiums. Nothing can be more encouraging to him in an occasional leisure moment than to examine his policy, note the number of premiums paid, the cash value the policy now has, the amount of money he could borrow from the company on the policy as security, and then to think of how small the effort has been to meet the payments. He will invariably thank Fortune that he had the prudence to insure when he did. In the late financial panic, when a member of Congress rose in his seat and asked, "Is there a man within the sound of my voice who can today, in any bank in America, borrow $1,000 on his personal note?" and no man answered, the life insurance companies were lending money to their policy holders, practically without limit. Banks and great commercial houses were failing on every hand, but not one of the great life insurance companies of this country failed.

Having decided that you want insurance, the question is, how much, and what kind of policy? and above all, in what company?

First, as to the company. You must keep in view the great cardinal truth of insurance, "The best is always the cheapest." Never buy insurance because it is cheap. You might as well buy cheap mining stock—a thousand chances to one it will prove worthless. Never buy insurance because of statements made by an insurance agent. Your lawyer, or some other friend, will lend you the report of the insurance commission of your State. You can there see the tables of assets of the various companies. You can then select five or ten of the oldest and strongest companies. You will find that they all charge about the same rate for the same form of policy, and that each claims to have some advantage in its favor over the others. Each claims that the insurance laws under which it is organized are drawn to protect the assured, to the possible detriment of the company. Maine and some other States have a "non-forfeiture" law, by which, if your policy continues in force for a stated period and you then lapse in your payments, the former payments will carry the policy for a greater or smaller period of time, proportioned to the amount paid in ; and so with other features of the policy.

Your choice from among these companies must be governed by your taste or necessities. Read the policy before you apply for insurance. Other things being equal, the policy that is shortest and simplest is the best. Reject the old-fashioned policy of blanket dimensions and covered with fine print. It may be a good policy for lawyers to fight over when you are dead, or want to cash in your insurance ; but brevity is the soul of wit in insurance, so far as the assured is concerned. Be sure you do not confuse the agent's talk with the terms of the policy. If he tells you that the company will do thus and so, ask him to show you the agreement in the policy. If it is not there, you may rest assured that the company will not do as he says. The twenty-payment life policy is the most popular form now issued, although other forms have advantages over it. Remember that the agent's commissions vary according to the kind of policy, and the kind he urges you to buy may be the best for him, but the poorest for you. There are many things in this connection of which much might be said. But if you insist on seeing a sample policy and reading it carefully before making your application, you cannot be duped. Be certain that the policy is written in plain, simple English, and is brief, and you will never have cause to regret buying it, unless you buy too large an amount.

And this brings us to the question of how much you ought to carry. This is a very practical question. You will find men who sneer at insurance. On investigation you will find that they are men who have invested a large sum in an insurance policy, out of all proportion to their incomes, and have left future payments to luck. The succeeding payment is not made and the policy lapses. The holder of the now worthless policy looks at his first premium receipt, and thinks the company has robbed him, and vows he will never be caught in a similar trap again. This sounds like nonsense, but it is literal truth in thousands of cases. Avoid extremes. Buy a small policy ; then another, of another kind if you like, so as not to have all your eggs in the same basket. The agent will not like this course, but you are investing for your own benefit and not his. A live policy for a $1,000 is worth a thousand lapsed policies aggregating a million.

And when you come to the experience that the great majority of physicians and lawyers meet at the end of a life of toil for others, seeing the end draw nigh, and the wife and little ones not otherwise provided for, your mind will be eased of a great burden as you think of the stray fees you have paid for your insurance. —*Medical Mirror.*

---

LIABILITY FOR INJURIES WHEN DEATH FOLLOWS SURGICAL OPERATIONS.—When death results from a personal injury, as its direct and proximate cause, the party responsible for the latter is liable for such death. In the case of Rettig vs. Fifth Avenue Transportation Company, recently decided by the Superior Court of New York City, General Term, which was an action brought to recover damages for alleged negligent acts causing injuries resulting in the death of the injured person, the defense was raised that death was rather caused by a surgical operation than by the injuries complained of. One of the attending physicians at the hospital to which the injured man was taken, testified that he died from the result of his injuries, that his condition necessitated a surgical operation, which was skilfully performed, and that he died of the shock that followed. This evidence is declared admissible, and the court holds that the surgical operation and the consequences flowing from it in no manner relieved the transportation company from liability for the death as a result of the injuries inflicted by its negligence. Indeed, the court shows that it has been held that when a person who, through the negligence of another, has received an injury which, without a surgical operation, would cause his death, employs a competent and skilful surgeon, by whose mistake the operation is not successful, and the patient die, the wrongdoer is not shielded from liability by the surgeon's error; and this although the operation is the immediate cause of the death.—*Medical News.*

---

RECTAL AFFECTIONS IN YOUNG CHILDREN.—It sometimes happens that a discharge, dysenteric in character, is excited in young children by the existence of a polyp in the rectum. Whenever a child passes blood at stool it is well to bear this thought in mind. Internal medication avails nothing in such cases. It is not essential that a history of protrusion should be given, for many polyps have their origin high up the rectum and the pedicle is not of sufficient length to allow the little tumor to protrude. The growth should then be removed.—*Mathews's Med. Quarterly.*

